# Immediate non-submerged implants with laser-microtextured collar placed in the inter-radicular septum of mandibular molar extraction sockets associated to GBR: Results at 3-year

**DOI:** 10.4317/jced.56277

**Published:** 2020-04-01

**Authors:** Renzo Guarnieri, Dario Di Nardo, Gianni Di Giorgio, Gabriele Miccoli, Luca Testarelli

**Affiliations:** 1 MD, DDS, Adjunct Professor Dept. of Dental and Maxillofacial Sciences, School of Dentistry, University La Sapienza, Rome, Italy; 2 DDS, PhD, Dept. of Dental and Maxillofacial Sciences, School of Dentistry, University La Sapienza, Rome, Italy; 3 DDS, PhD, Associated Professors, Dept. of Dental and Maxillofacial Sciences, School of Dentistry, University La Sapienza, Rome, Italy

## Abstract

**Background:**

The aim of the present study was to radiographically evaluate the vertical socket walls changes, and the peri-implant marginal bone remodelling, and clinicallly the soft tissues conditions around the non-submerged single implants placed into the inter radicular septum of mandibular molar sockets, associated with a collagen membrane, after 3 years of loading.

**Material and Methods:**

Thirty patients underwent to placement of a non-submerged implants with a laser-microtextured collar into the inter radicular septum of mandibular molar fresh extraction sockets. A collagen membrane and the mucoperiosteal flap were adapted around the neck of the implants, leaving the laser-microtextured collar to heal in a transmucosal fashion.

**Results:**

At the end of the follow-up period, no statistical differences were found for each radiographic measurements used for the examination of extraction sockets vertical bone changes. Compared to implants placement, at the end of the 3-year follow-up, the vertical radiographic mesial and distal peri-implant marginal bone levels showed a statistically significant gain of 0.9 (SD 0.5), and 1.0 mm (SD 0.6), respectively (*P*=0.037).

**Conclusions:**

In mandibular fresh extraction sockets, the method of GBR around transmucosal implants with laser-microtextured surface placed into the interadicular septum may be used successfully to counteract the ridge remodelling.

** Key words:**Non-submerged implants, GBR, laser-microtextured collar.

## Introduction

Several literature data indicated that, with careful patient selection, the immediate implants placement protocol in molar extraction sockets has long-term success rate comparable to that of implants placement into healed molar extraction sites (1-4). One of the most important factors that has been considered essential for successful immediate implant placement in fresh molar extraction sockets is the primary implant stability (5). The anatomical width of molar sockets, the poor bone quality of posterior jaws (especially of the maxilla), and the poor availability of apical bone due to presence of the mandibular nerve and the maxillary sinus, often make it difficult to achieve a sufficient initial implant stability. To overcome these limitations, wide-diameter implants have been proposed (6-8). Increasing the surface area and the bone-to-implant contact area, wide-diameter implants can be beneficial in enhancing primary implant stability in sites with poor bone quality and poor availability of apical bone (9,10). However, especially in mandibular molar extraction sockets, which present a narrower bucco-lingual than mesio-distal space, the wide-diameter-implant/alveolar bone maximum contact at the vestibular and lingual wall, and the related high compressive stress, may result in microcracks and bone resorption, which may subsequently lead to early implant failure (10). To achieve primary stability in molar fresh extraction sockets, an alternative to wide-diameter implants is the placement of standard diameter implants into the inter-radicular septum (11). In these cases, since bone completely surrounds the implant, the remaining root spaces don’t need regenerative therapy for the implant to integrate. However, the socket spaces adjacent to the inter-radicular septum would needed be regenerate to reduce ridge remodeling and to achieve better aesthetics and prosthetic contours (6). Though guided bone regeneration (GBR) was thought to be possible only associated to submerged immediate implants, several reports highlighted that a GBR technique can be used with success associated with immediate non-submerged implants (12-15), also with the concomitant aim to counteract the ridge remodeling (16,17). In these situations, if the bony borders of the sockets can adequately support the membrane, compared to those not reabsorbable, reabsorbables membranes present several advantages, such as, an improved soft tissue healing, the incorporation by the host tissues, and a quick resorption in case of exposure (18-20).

A problem often encountered when placing implants associated to membranes immediately into extraction sockets, is a lack of soft tissues for primary healing. Conflicting results have been reported regarding the amount of bone regeneration that occurs in the presence of secondary wound and membrane exposition. Some investigators have reported compromised results (21,22), while others still obtained very good defect fill with new bone (14,23). As regards the not submerged implants, these results appear to be at least partly caused by the closure, or not, of soft tissues against the implant surface, thus covering the membrane during the regenerative period (24).

Numerous histological reports in human and animal have been published documenting an intimate physical contact between a laser-microgrooved implant collar surface and peri-implant soft tissues (25,26). Laser-produced microgrooves are a series of cell-sized circumferential isotropic channels onto the titanium surface at a coronal height of the implant collar, which act as a predetermined site, attracting the formation of a physical connective tissue, with fibers perpendicularly oriented to the implant collar surface. Histological research in animal (27) have compared the healing process after immediate implantation of microgrooved and smooth collar implants in fresh extracted sockets. In the microgrooved group, the collagen fibers showed a perpendicular orientation to the implant collar surface, whereas in the turned surface group, the fibers were parallel to the fixtures. In addition, in the microgrooved groups, the epithelium migrated down to where the connective tissue was attached, whereas in the turned surface group, the epithelium grew downward to where the thread began passing over the turned surface.

The present study was designed to evaluate whether a method of GBR associated to immediate transmucosal implants could be particularly beneficial combining a resorbable membranes to a laser-microgrooved implant collar surface. For this purpose, the peri-implant marginal bone changes and soft tissues conditions around the non-submerged single implants placed into the inter radicular septum associated with a collagen membrane, after 3 years of loading, were investigated.

Clinical results of horizontal hard and soft tissue changes, and radiographic results of vertical socket walls and the peri-implant marginal bone remodelling before implants loading have been previously reported (28). The present paper reports results after 3 years of implants loading.

## Material and Methods

Patient selection: Thirty patients, who required implant therapy for the replacement of mandibular hopeless molar teeth, were identified and enrolled in this study. Criteria for inclusion were: age ≥ 18 years, good general health, presence of molar extraction socket type 1 according with the classification suggested by Juodzbalys *et al.* (29), and a presence of inter radicular septum with a sufficient amount of bone to place a standard implant (3.8 mm diameter and 9 mm length), detecTable by means of CBCT evaluation. Exclusion criteria were: natural teeth adjacent to surgical area affected by untreated periodontal or endodontic infections, absence of opposing occlusion, full-mouth plaque score (FMPS) ≥25%; full-mouth bleeding score (FMBS) ≥25% recorded at the time of implant placement, para-functional habits, severe maxilla-mandibular space discrepancies, uncontrolled diabetes, treatment with bisphosphonates, patients smoking >10 cigarettes a day, and any drug/alcohol abuse. All patients were informed about the evidence-based, positive outcome of the immediate implants placement approach associated with GBR technique that were tested. Each patient signed a free informed consent form after he/she has received detailed information about the study. Treatments were performed according to the principles outlined in the Declaration of Helsinki on experimentation involving human subjects.

Implants: Thirty non-submerged implants (BioHorizons Tissue Level Laser-Lok®, Birmingham, Al, USA) were used for the study. Implants have the body grit-blasted to create a moderately rough surface, while the apical 2.0 mm of the collar are characterized by the presence of laser-produced microgrooves on the range of 8μm, and the most coronal 0.3mm of the collar is smooth, machined metal.

**Figure 1:**
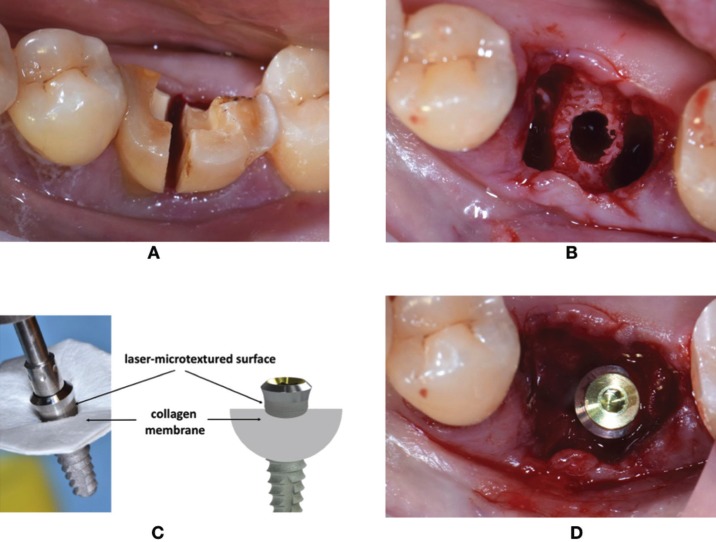
A) The tooth was sectioned to make the extraction the least traumatic possible. B) preparation of the inter-radicular recipient site. C) before the positioning, the membrane is cropped according to the measurements of the post-extraction socket perimeter, and the implant is inserted into the center of the membrane exactly in the transverse area between the surface of the implant body and the laser-microtextured collar. D) Simultaneous implant and membrane placement. The membrane is sustained in the center by the implant, and along its perimeter by the extraction socket walls, leaving the laser-microtextured collar in contact with the soft tissue.

Surgical procedure: All implants were placed by the same operators (RG, LT). All the subjects adopted an antimicrobial prophylaxis with mouthrinses of 0.12% chlorhexidine 1-minute rinse before surgery and three times a day for the following 10 days (Dentosan 0.12%, Johnson & Johnson, USA). Amoxicillin + clavulanic acid 1 g bid (Augmentin, Glaxo SmithKleine, Italy) was prescribed for 7 days. Local anesthesia was induced by infiltration with articaine/epinephrine (1:100.000) (Ecocain 20mg/ ml, Molteni Dental, Italy). Each mandibular molar tooth, if necessary, was sectioned to make the extraction the least traumatic possible, and a flapless procedure was performed for the extraction (Fig. 1A). The preparation of the inter-radicular recipient site was performed following the instructions of implant manufacturer under abundant saline solution irrigation (Fig. 1B). A collagen membrane (Mem-Lok Pliable®, BioHorizons, Birmingham, AL, USA) was used in each molar extraction socket. Mem-Lok Pliable® is a porcine- derived resorbable collagen-based membrane with an estimated resorption time of 12-14 weeks. Before the positioning, the membrane was cropped according to the measurements of the post-extraction socket perimeter, and the implant was inserted into the center of the membrane exactly in the transverse area between the surface of the implant body and the laser-microtextured collar (Fig. 1C). In this way the simultaneous implant and membrane placement allows to position the laser-microtextured implant collar above the inter-radicular septum, and the membrane above the lingual/palatal and vestibular bone crest. Before the simultaneous implant and membrane placement, the interdental mesial and distal papilla was prepared with a pouch procedure, to allow, mesially and distally, the placement of the membrane under the interdental papilla. In this way, the membrane is sustained in the center by the implant, and along its perimeter by the extraction socket walls, leaving the laser-microtextured collar in contact with the soft tissue (Fig. 1D). Care was exercized to extend the membrane at least 3 mm beyond the bony edges of the defeacts.

Sutures were used to stabilize the membrane. Patients were instructed to have a liquid or semiliquid diet for the first three days and then gradually return to a normal diet. An analgesic (Ibuprofen®, 600 mg) immediately after the surgical intervention and after 8 hours were prescribed.

Sutures were removed 2 weeks post-implant insertion. After 4/6 months, impression were taken, definitive prosthetic abutments were connected, and definitive crowns were delivered.

Radiographic examination: Radiographs were performed immediately at the implant placement (T0), 4 months after surgery (T1), at the delivery of definitive crowns (T2) and at the end of follow-up period, after 3 years of implant loading (T3), with a paralleling technique using a Rinn film holder with a rigid film-object X-ray source. For the radiograph procedures, a silicone index material was fixated to the residual dentition and a radiograph holder was constructed for each patient. This technique ensured that the same position of the radiograph film could be reproduced at each visit and the angle of the radiograph would not deviate. The radiographs were taken in high resolution mode (Vista Scan Durr Dental, Durr Dental Italy S.r.l) with a dental x-ray machine (TM 2002 Planmeca Proline CC, Planmeca Group Helsinki, Finland) equipped with a long tube that operated at 70 Kw/7.5 mA. Specialized software (DBSWIN software, Durr Dental Italy S.r.l) was used for linear measurements of marginal bone changes. The following radiographic measurements were performed:

- radiographic implant length (IL): distance (in mm) between the implant shoulder and the implant apex as assessed at the mid portion of the implant;

- residual bone height at the mesial (MI) and distal (DI) aspects of the implant: distance between the line linking the coronal implant margin, and the first contact of the crestal bone on both mesial and distal sides of the implant.

- bone height at the mesial (MM) and distal (MD) aspects of the residual mesial extraction socket bone peak, and bone height at the mesial (DM) and distal (DD) aspects of the residual distal extraction socket bone peak, measured in mm from the line linking the CEJs of the adjacent teeth.

To account for radiographic distortion, radiographic measurements on each radiograph were adjusted for a coefficient derived from the ratio: true length of the implant/IL. For each implant, the MBL was calculated as the mean value of MI and DI. All measurements were carried out by a single trained examiner who had previously undergone a calibration session for radiographic assessment on a sample of 10 patients treated with the same implant system and not included in the study (Kappa Test= 0.940, SE of kappa = 0.042, 95% confidence interval: from 0.857 to 1.000). In Figure is showed an example of radiographic measurements used for the evaluation.

Clinical examinations: At the sutures removal (10-15 days), and at 4, 8, and 12 weeks of healing, the conditions of the soft tissues at the treated sites were evaluated. The presence of flap dehiscence and/or recession around the implant collar, and/or suppuration was assessed. At each year control visit, the following clinical parameters were assessed: FMPS, FMBS, probing depth (PD), bleeding on probing (BOP) and gingival recession (REC). Probing depth and bleeding on probing were recorded at four sites around each implant (mesial, buccal, distal, and oral) using a graduated manual periodontal probe.

Statistical analysis: statistical analysis was performed using 13.0 SPSS® statistical program (SPSS, Chicago, IL, USA). Results were expressed as mean, standard deviation, median, and range. Data were analyzed by means of Mann–Whitney test. A *P* value < 0.05 was considered statistically significant.

## Results

**Figure 2:**
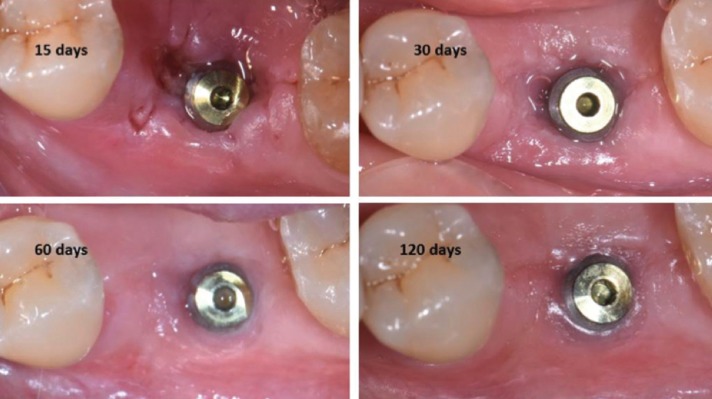
Example of a soft tissues healing.

In Table 1 are reported the patient population and the mean values of clinical index evaluated during the follow-up. At the sutures removal 88% of flaps showed primary closure. After 4, 8 and 12 weeks of healing, 92%, 97% and 100%, respectively, of sites were completely healed. Flap dehiscence was recorded in 8% of sites at the sutures removal. At the 4-, 8-, 12-week examination, flap dehiscence was noted in 4%, 2%, and 0% of sites, respectively. In Figure 2 is reported an example of the soft tissues healing during the 2, 4, 8, and 12 weeks.

**Table 1:**
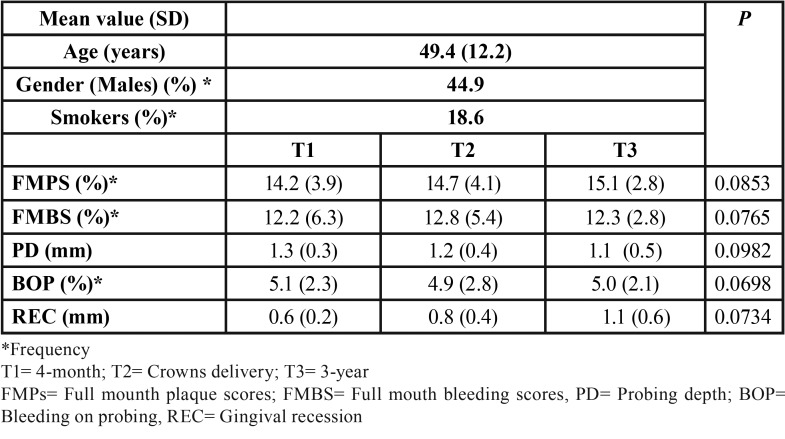
Patient population and mean values of clinical index evaluated during the follow-up.

**Figure 3:**
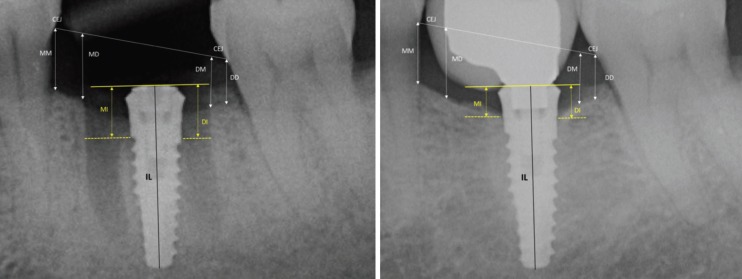
Example of the radiographic examination at the implant and membrane placement (T0) and at the end of the 3 years follow-up post-loading (T3). MM, MD = bone height at the mesial and distal aspects of the residual mesial extraction socket bone peak; DM and DD bone height at the mesial and distal aspects of the residual distal extraction socket bone peak, measured in mm from the line linking the CEJs of the adjacent teeth. MI and DI= peri-implant marginal bone mesial and distal levels.

The overall survival rate from baseline to the 3-year follow-up visit was 100%. Mean MBL values are reported in Table 2. At the end of the follow-up period (T3), no statistical differences were found for each radiographic measurements (MM, MD, DM, and DD) used for the examination of extraction sockets vertical bone changes (*p* >0.05). Compared to T0 (implants placement), at the end of the 3-year follow-up, the vertical radiographic mesial and distal marginal bone levels showed a statistically significant gain of 0.9 (SD 0.5), and 0.10 mm (SD 0.6), respectively (*P*<0.05). (Fig. 3, Table 2).

**Table 2:**
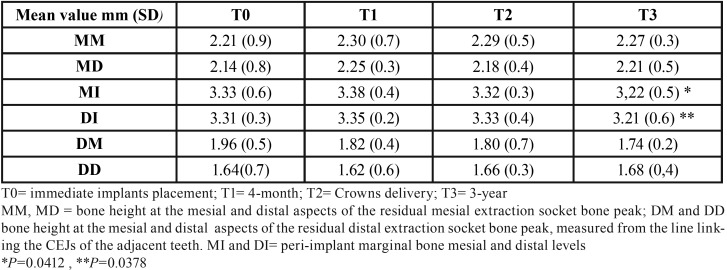
Radiographic measurements evaluated during the follow-up.

## Discussion

The present paper reports after 3 years of follow-up, clinical and radiographic results of a cases series study, aimed to evaluate whether a method of GBR could be particularly beneficial combining a resorbable membrane to a non-submerged immediate implant with a laser-microgrooved collar surface, placed into the mandibular molar interadicular septum. The previously reported data (28), collected before implants loading, have indicated no statistically significant difference in the early MBL between the radiographic vertical measurements taken at implant placement, compared to that recorded after 4 months of healing. Moreover, no statistical differences were also founded for each radiographic measurements used for the evaluation of extraction sockets vertical bone changes. These results had showed that the clinical method of GBR performed in the current study, may be used successfully to counteract the ridge remodelling and the early MBL around transmucosal implants placed into fresh mandibular molar extraction sockets. Data collected after 3 years of loading showed no statistical differences for each radiographic measurements (MM, MD, DM, and DD) of socket walls vertical changes. They confirms the previous published results and indicate that GBR technique with collagen membrane supported in its perimeter by the residual post-extractive alveolar walls and in the center by the transgingival emergence of the implant, presents sTable results after 3 years of implants loading.

These outcomes are in agreement with results of several studies indicating that an high predictability of a simultaneous GBR technique can also be achieved with a one-step trans-mucosal-healing approach (4-7). Results reported by of surgical re-entry evaluations in sites treated with GBR and non-submerged implants indicated a mean bone fill between 75% and 94% (24).

However, it is important to stress that GBR technique associated to non-submerged implants does not provide a primary closure of the flap completely covering both membrane and implant. In these cases, the flap is adapted around the collar of the implant, covering the membrane but leaving the implant in transmucosal position. It has been previously claimed that primary wound closure following GBR shoud be a prerequisite for the formation of new mineralized bone (30,31). This statement is based on the finding that new bone formation was less favorable when dehiscences occured, compared when soft tissues remained intact above the membrane (24). As regards the not-submerged implants associated with GBR, the flap dehiscence, connected with the transmucosal implant position, could represent a complication usually leading to a compromised healing outcomes.

Histological studies on human and animal showed that a laser-microtextured surface on implant collar allows the formation of a physical soft tissue attachment with perpendicular/ functional organization of connective tissue fibers, both in native bone, and in post-extractive fresh extraction sockets (32,33). Recent gene profiling analysis documented that the mucosal wound healing around dental implants is influenced by the topographic nature of the collar surface (34). In biopsies obtained after 2, 4, and 8 weeks, at the laser-microtextured vs. machined implant collar surface, a differential gene expression was revealed. mRNAs encoding keratins and protective proteins of cornified epithelium were upregulated in tissues from laser-modified surfaces. Moreover, in tissue from laser-modified surfaces, upregulation of mRNAs encoding proteins associated with collagen fibril formation and function was observed at 4 weeks. These data suggested that a micro-scale laser-microtextured surface mediates alterations in the junctional epithelium component, in integrin receptors and extracellular matrix protein expression, which contribuite to modulation of the process of peri-implant/mucosa integration.

The GBR technique used in the current study allows at the same time leaving free the laser-microtextured collar above the reasorbable membrane totally in contact with the soft tissue of post-extractive site during the healing phase, while the membrane covering the extraction socket’s mesial and distal alveolar bone defects. Measurements of mesial and distal peri-implant marginal bone levels, after 3 years of loading, showed a statistically significant bone gain with a average of 0.10 mm (Fig. 3). A possible explanation of this results could be linked with the abilty of the laser-microtextured collar surface to create a soft tissue seal that counteracts the downgrouth of epithelium, and protects the underline bone from the oral environment. The MBL that occurs after implant placement is related to the biologic width associated with implants (35). An histological study in dogs by Shin & Han (33) compared the MBL after immediate implantation of laser-mocrotextured and smooth collar implants in fresh extracted sockets. Results do¬cumented that at the 12-week interval, the mean bone–implant contact in the laser- microtextured group was significantly higher than that of the turned surface group (about 60% vs. 40%). Moreover, in the laser-microtextured groups the epithelium migrated down to where the connective tissue was attached to the collar implant surface, whereas in the turned surface group, the epithelium grew downward to where the thread began passing over the turned surface. Epithelial downgrowth on titanium surfaces is attribuTable to coronal–apical proliferation and migration of epithelial cells derived from the mucosa surrounding the wound surface forming a junctional epithelium of a thickness of about 2 mm (36). The presence of granulation tissue in contact with the transmucosal titanium surfaces is thought to be one of factors favoring apical epithelial migration, and the related early MBL (37). Material properties appear to be another factor affecting epithelial downgrowth. Kim *et al.* (38) compared the effects of abutment shapes relative with MBL. They compared implants with micro-textured transmucosal profiles, machined profiles, and straight anodically oxidized profiles. Around machined and anodically oxidized profiles, the junctional epithelium was found longer, around laser-microtextured profiles epithelium was shorter, connective tissue attachment was more extended and the bone-level stable. The location of the junctional epithelium appears to be determined by the initial phases of wound healing (39) and by the structural conformation of the connective tissue. (40,41). According with the results of the present study, it is possible to hypothesize that peri-implant marginal bone gain recorded during the healing and implants function phase, could be linked to maintaining of the vestibular and lingual alveolar walls dimensions and of the mesial and distal bone peaks, and to soft tissue healing process around the laser-microtextured collar surface, with the formation of peri-implant soft tissue seal.

Exposure of membranes and infection seem to be common findings associated with GBR at immediate transmucosal implants (24). In all examinated patients, after the suture removal (15 days) only 12 % of the membrane exposure, without infection, was noted. These findings could be associated with the chemical/physical features of the membrane used in the present study (MemLok Pliable). *In vitro* and in animal analysis (42) has been documented that the MemLok membrane elicits a low inflammatory and foreign body giant cell response, suggesting that the chemical treatments of the membrane have reduced the extent of inflammation and foreign body reactions. The low degree of inflammation and foreign body response may result in enhanced tissue integration and improved wound healing in terms of minimizing scar-like tissue formation.

## Conclusions

From the results of the present study, it is concluded that in mandibular fresh extraction sockets, the method of GBR around transmucosal implants with laser-microtextured surface placed into the interadicular septum, seems be particularly beneficial when the combination of implantation and resorbable membranes can eliminate the need for a second surgical procedure.
